# Drinking Water in California Child Care Sites Before and After 2011–2012 Beverage Policy

**DOI:** 10.5888/pcd12.140548

**Published:** 2015-06-04

**Authors:** Lorrene D. Ritchie, Sallie Yoshida, Sushma Sharma, Anisha Patel, Elyse Homel Vitale, Ken Hecht

**Affiliations:** Author Affiliations: Sallie Yoshida, Sarah Samuels Center for Public Health Research and Evaluation, Oakland, California; Sushma Sharma, Atkins Center for Weight and Health, University of California, Berkeley, California; Anisha Patel, School of Medicine, University of California, San Francisco, California; Elyse Homel Vitale, California Food Policy Advocates, Oakland, California; Ken Hecht, Nutrition Policy Institute, Division of Agriculture and Natural Resources, University of California, Oakland, California. Dr Sharma is now affiliated with Dallas-Fort Worth Hospital Council Foundation, Irving, Texas.

## Abstract

**Introduction:**

Drinking water is promoted to improve beverage nutrition and reduce the prevalence of obesity. The aims of this study were to identify how water was provided to young children in child care and to determine the extent to which water access changed after a federal and state child care beverage policy was instituted in 2011 and 2012 in California.

**Methods:**

Two independent cross-sectional samples of licensed child care providers completed a self-administered survey in 2008 (n = 429) and 2012 (n = 435). Logistic regression was used to analyze data for differences between 2008 and 2012 survey responses, after adjustment for correlations among the measurements in each of 6 child care categories sampled.

**Results:**

A significantly larger percentage of sites in 2012 than in 2008 always served water at the table with meals or snacks (47.0% vs 28.0%, *P* = .001). A significantly larger percentage of child care sites in 2012 than in 2008 made water easily and visibly available for children to self-serve both indoors (77.9% vs 69.0%, *P* = .02) and outside (78.0% vs 69.0%, *P* = .03). Sites that participated in the federal Child and Adult Care Food Program had greater access to water indoors and outside than sites not in the program. In 2012 most (76.1%) child care providers reported no barriers to serving water to children. Factors most frequently cited to facilitate serving water were information for families (39.0% of sites), beverage policy (37.0%), and lessons for children (37.9%).

**Conclusion:**

Water provision in California child care improved significantly between samples of sites studied in 2008 and 2012, but room for improvement remains after policy implementation. Additional training for child care providers and parents should be considered.

## Introduction

On any given day, more than one-quarter of young children in the United States do not drink plain water (1) and some children may not be adequately hydrated (2). Inadequate hydration can impair cognitive and physical functioning (3–5). Consuming sugar-sweetened beverages for hydration may put children at risk for obesity (6), whereas substituting plain, zero-calorie water for caloric beverages may reduce energy intake (7) and weight (8). As the prevalence of pediatric obesity has steeply risen in recent decades, children’s intake of sugar-sweetened beverages has increased and water intake has been low (9,10). Accordingly drinking water was identified by the Centers for Disease Control and Prevention as critical for improving population nutrition and reducing obesity (11).

Currently several policies exist to support healthy beverages as part of federal obesity prevention efforts (12). As mandated by the federal Healthy, Hunger-Free Kids Act (HHFKA) of 2010, free drinking water must be available to students at lunchtime in schools that participate in the National School Lunch Program (13). Similarly, as of October 2011, drinking water must be available to children throughout the day, including at meal times, in child care sites that participate in the federal Child and Adult Care Food Program (CACFP) (14), the child care equivalent of the federal school nutrition programs. Numerous states also have enacted legislation to improve water access in child care settings (15). The California Healthy Beverages in Childcare Law, enacted in 2010 and implemented beginning January 2012, extends water provision to all licensed child care settings to ensure that drinking water is readily available throughout the day, including at meals and snack-times (16).

Although early childhood is an optimal time to establish beverage behaviors that track into later life (17,18), surprisingly little is known about water provision in child care settings. In 2008, before the federal and state policies were enacted, we conducted a statewide survey of child care providers in California to investigate the beverages served to children aged 2 to 5 years in child care. In 2012 we repeated the survey after both the federal and state policies on water provision went into effect. Using data from 2008 and 2012, the primary aims of this study were to 1) compare water access before and after the policies were enacted; 2) compare differences in water provision by type of child care in 2012; and 3) describe how water was provided to young children in child care and identify barriers and facilitators to serving water in 2012.

## Methods

### Design

Two independent cross-sectional samples of licensed child care providers in California completed a self-administered survey (paper or online) in 2008 and 2012. All procedures involving human participants were approved by the Committee for the Protection of Human Subjects at the University of California, Berkeley.

### Sample selection

Identical methods were used in 2008 and 2012 as described previously (19). A stratified random sample of 1,484 child care sites was selected in late 2011 from state databases of all (over 50,000) licensed child care settings in California. Six strata of child care settings were examined on the basis of 2 factors presumed to influence the beverage environment: whether the setting is a center or family home and whether or not a participant is in CACFP. An approximately equivalent number of sites was selected in both 2008 and 2012 from each strata: Head Start centers (CACFP participation required), state preschools (required to follow CACFP nutrition standards but can choose whether to participate in CACFP or the National School Lunch Program), other centers participating in CACFP, non-CACFP centers, family child care homes participating in CACFP, and non-CACFP family child care homes.

### Survey instrument

Survey questions were adapted from a previous survey (20) or newly developed for study aims and are available online (21). New survey questions were reviewed by experts for content validity and pretested for readability, comprehension, and length of completion with staff of child care sites participating in another research project. Surveys were translated into Spanish by a bilingual research staff member and checked for accuracy and readability by a second bilingual research staff member; discrepancies in translation were discussed and resolved. Each survey took approximately 20 minutes to complete.

In both 2008 and 2012, questions were asked about how often drinking water was provided to children at the table with meals or snacks and about the availability of self-serve drinking water indoors and outside. Included in the 2012 survey only was a frequency checklist, which asked respondents to record the types of water served to children aged 2 to 5 years on the day preceding the survey. Types of water included plain bottled water (no added flavors or sweeteners); bottled water with flavors, vitamins, or sweeteners added; and tap water. Respondents were instructed to include water provided by the child care site as well as water brought by parents and to indicate whether served at a meal (breakfast, lunch, dinner) or a snack. In 2012, additional questions also were asked about the site’s source of water, how drinking water was made available to children both indoors and outside, and factors influencing water provision.

### Sample recruitment and data collection

Methods were the same in 2008 and 2012. Selected child care sites were mailed postcards, in English and Spanish, inviting 1 or more staff familiar with foods and beverages served at the site to complete an online survey. A reminder letter, a hard copy of the survey, and a stamped return envelope were mailed 2 months later to nonrespondents. Up to 3 follow-up telephone calls were made to unresponsive sites approximately 1 month after reminder postcards were sent. Family home providers could complete the survey in English or Spanish.

### Data analysis and statistical power

Of the 456 completed surveys in 2012 (30% response), 8 were excluded as sites caring only for children under 2 years old and 13 were excluded for incomplete data on site characteristics and beverages served. In 2008 the response rate was similar (31%). Of family home providers, 12% in 2012 and 16% in 2008 completed the survey in Spanish. No more than 5% of responses were missing for any survey item; imputation of missing data was not performed and nonresponses were not included in the denominator. Because we were interested in comparing child care categories rather than obtaining population estimates, sample weights were not used.

Data were analyzed using SAS version 9.3 (2011, SAS Institute, Inc). Differences between categories were determined by using χ^2 ^tests or logistic regression for categorical variables and analysis of variance for continuous variables. Water provision was compared by the 6 categories of child care sites, by CACFP participation, and whether a child care center or family day care home. Adjustments for multiple comparisons were made using the Tukey-Kramer test. Binary measures (ie, whether or not water was provided) were created and logistic regression was used to analyze for differences between 2008 and 2012, adjusting for differences between each category (ie, Head Start, state preschool, other CACFP center, non-CACFP center, CACFP home, non-CACFP home). A significance level *P* < .05 was used for all statistical tests.

## Results

The final sample consisted of 429 child care sites in 2008 and 435 child care sites in 2012, representing data on 31,990 children in child care in California in 2008 and 34,413 in 2012 (Table 1).

**Table 1:** Sample Sizes for Study of Licensed Child Care Providers in California, 2008 and 2012

Category	2008 Sample (n = 429)	2012 Sample (n = 435)
**Centers**	326	301
Head Start centers participating in CACFP	66	78
State preschools participating in CACFP or the equivalent	68	93
CACFP centers	104	48
Non-CACFP centers	88	82
**Family day care homes**	103	134
CACFP homes	65	93
Non-CACFP homes	38	41
**Children aged 2 to 5 y**	31,990	34,413

Abbreviation: CACFP, Child and Adult Care Food Program.

**Figure 1:**
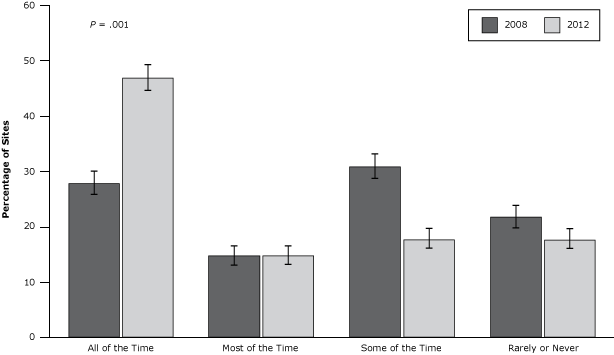
Frequency of providing drinking water at the table with meals or snacks in 2008 and 2012. Logistic regression adjusted for correlations among measurements in each of the 6 child care categories (Head Start, state preschool, other CACFP center, non-CACFP center, CACFP home, non-CACFP home). Sums of responses for each year are slightly less than 100% (96% for 2008; 98% for 2012) because of missing responses. Standard error bars are shown. Abbreviation: CACFP, Child and Adult Care Food Program. **Table:** 

In 2012 child care sites were 2.36 times more likely to provide water at meals or snacks than sites in 2008 (95% confidence interval [CI], 1.75–3.13; *P* = .001). A larger percentage of sites served water all of the time at the table with meals or snacks in 2012 than in 2008 (47.0% vs 28.0%, *P* = .001) (Figure 1). We found no differences in the percentage of sites always serving water at the table between CACFP and non-CACFP sites or between centers and homes.

Frequency	2008, % (SE)	2012, % (SE)
All of the time^a^	28.0 (2.2)	47.0 (2.4)
Most of the time	14.9 (1.7)	14.9 (1.7)
Some of the time	31.0 (2.2)	17.9 (1.8)
Rarely or never	21.9 (2.0)	17.9 (1.8)

Abbreviation: SE, standard error.

^a^ *P* = .001 for difference between 2008 and 2012.

In 2012 nearly half (47.6%) of providers reported that children were allowed unlimited self-serve water, whereas 20.5% of sites provided water only upon request by children. A total of 5.8% provided water only after children had finished their milk or juice, whereas 4.1% provided water only after children finished their meal or snack.

A larger percentage of child care sites made water easily and visibly available for children to self-serve both indoors (77.9% vs 69.0%; odds ratio [OR] = 1.47, 95% CI, 1.08–1.98, *P* = .02) and outside (78.0% vs 69.0%; OR = 1.59, 95% CI, 1.17–2.17, *P* = .03) in 2012 compared with 2008. In 2012, more CACFP than non-CACFP sites had water easily available indoors and outside (*P* = .03). Although most centers (73.1%) made water easily available to children to serve themselves indoors and outside in 2012, less than half of homes (44.8%) reported doing so (*P* < .001).

**Figure 2:**
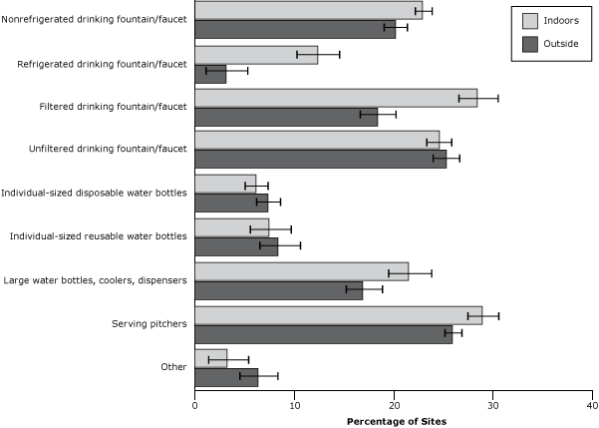
Methods used to make drinking water available for children indoors and outside the site in 2012. Respondents were asked to check all answer options that applied so responses for indoors and outside sum to more than 100%. These data were not collected from child care providers in the 2008 sample. Standard error bars are shown. **Table:** 

Various methods were used by child care sites in 2012 to make water available for children indoors and outside. Nonrefrigerated fountains or faucets; filtered or unfiltered fountains or faucets; large bottles, coolers, or dispensers; and serving pitchers were the most commonly reported; individual-sized disposable and reusable water bottles were less common (Figure 2).

Method	Outside, % (SE)	Indoors, % (SE)
Other	6.4 (1.9)	3.4 (2.0)
Serving pitchers	26.0 (0.8)	29.0 (1.6)
Large water bottles, coolers, dispensers	17.0 (1.9)	21.6 (2.2)
Individual sized reusable water bottles	8.5 (2.1)	7.6 (2.1)
Individual sized disposable water bottles	7.4 (1.3)	6.2 (1.2)
Unfiltered drinking fountain/faucet	25.3 (1.3)	24.6 (1.3)
Filtered drinking fountain/faucet	18.4 (1.8)	28.5 (2.0)
Refrigerated drinking fountain/faucet	3.2 (2.1)	12.4 (2.2)
Non-refrigerated drinking fountain/faucet	20.2 (1.2)	23.0 (0.9)

Abbreviation: SE, standard error.

In 2012 most sites (71.0%) provided tap water, and approximately half served bottled water (Table 2). More centers than family day care homes provided tap water (*P* = .01). Although more CACFP sites than non-CACFP sites tended to provide all types of water at meals and snacks, none of the differences were significant.

**Table 2:** Child Care Categories Serving Different Types of Water at Any Meal or Snack on the Day Before the Survey and Comparisons by CACFP Participation and Whether a Child Care Center or Family Day Care Home, 2012^a^

Water Type^b^	Child Care Category^c^, %							Comparison^d^	
	All (n = 435)	Head Start (n = 78)	State Preschool (n = 93)	CACFP Center (n = 48)	Non-CACFP Center (n = 82)	CACFP Home (n = 93)	Non-CACFP Home (n = 41)	CACFP vs non-CACFP^b^ (*P* Value)	Center vs Home^b^
Any type	91.0	82.1	87.1	79.2	93.9	86.0	92.7	CACFP > Non-CACFP (.08)	Center < Home (.09)
Bottled, plain	43.4	36.0^e^	21.5^f^	29.2^e^^, ^ f	47.6^g^	67.7^h^	61.0^g^^, ^ h	CACFP > Non-CACFP (.08)	Center < Home (.07)
Bottled, flavors, vitamins, or sweeteners added	14.9	14.1^g^	9.7^f^	10.4^e^^, ^ f	12.2^e^	19.4^g^^, ^ h	26.8^h^	CACFP > Non-CACFP (.10)	Center < Home (.09)
Tap	71.0	76.9^e^	86.0^f^	77.1^e^	73.2^e^^, ^ f	53.8^g^	53.7^g^^, ^ h	CACFP > Non-CACFP (.07)	Center > Home (.01)

Abbreviation: CACFP, Child and Adult Care Food Program.

a Data were not collected from child care providers in the 2008 sample.

b Providers were asked to mark all types of water served among the following options: plain bottled, flavored or sweetened bottled, tap or faucet.

c Values that do not share a common superscript (e, f, g, or h) across a row are significantly different by Tukey-Kramer adjustment for multiple comparisons, whereas values that do share a common superscript are not significantly different.

d Comparisons by logistic regression.

When providers were asked in 2012 if they ever use additions to water to encourage children to drink water, most indicated they did not; plain water was the most popular water choice. A minority (17.0%) used additions such as fruit slices, fruit juice, sugar-free powders, or other ingredients such as herbs; no sites reported adding sugar powders.

In 2012 most child care providers reported no barriers to serving water to children (76.1%). A minority reported issues that made provision of water difficult, including a perceived government rule against serving water with meals and snacks (3.4%); lack of CACFP reimbursement for water (3.0%); unavailability of water in site locations (indoors or outside) (3.0%); cost of bottled water, filters, or cups (2.1%); and concerns that children will drink less milk or eat less food if served water (2.1%). Less than 1% of providers agreed that any of the following were barriers to serving water: bad taste of water, concern about fluoridation, water safety concerns, environmental impacts (eg, use of disposable cups or bottles), children’s dislike of drinking water, concern that children would need to use the restroom more often, or lack of enough drinking fountains or faucets on site. Most providers (76.1%) did not know the source of the tap water at their site; 14.9% indicated ground water, 3.9% indicated a municipal water source, and 5.1% did not respond to this question.

The 3 factors most frequently cited by providers that facilitate serving more water to children were information for families (39.0% of sites), beverage policy (37.0%), and lessons for children (37.9%). Other factors cited as helping with increased water provision were parent and family support (27.1%) and training for child care providers (23.0%).

## Discussion

This is the first study to conduct an evaluation of the impact of federal and state drinking water policy in child care in the United States. The CACFP regulation states that “Throughout the day, including at meal times, water should be made available to children to drink upon their request, but does not have to be available for children to self-serve” (19). The California beverage policy specifies that all licensed child care “make clean and safe drinking water readily available and accessible for consumption throughout the day” (21). We chose to focus on availability of water for children to self-serve indoors and outdoors in addition to water provision with meals and snacks, on the basis of the assumptions that child care providers have limited time to serve water to children individually upon request and that children aged 2 to 5 years may not ask for water even when thirsty. Neither policy prescribes these practices. After policy implementation, we found significant improvement in the proportion of sites that always served water at the table with meals or snacks; almost half of providers offered self-serve water at the table. Significant improvement was also found in the proportion of sites that made water easily and visibly available for children to serve themselves indoors and outside. Lastly, sites used various methods for making water available, with filtered fountains, faucets, and pitchers being the most common indoors and outdoors.

Few studies of water availability in child care sites have been conducted. In a study of 40 CACFP centers in Connecticut conducted before the HHFKA, 84% had water accessible in classrooms and one-third had water accessible during physical activity periods (inside or outdoors) from a combination of adult-accessible and child-accessible sources; water was not served at the table at any of the lunches (1). In our study, before beverage policy we found a slightly lower proportion of sites (69%) that had water easily available for self-service indoors. Our inclusion of CACFP and non-CACFP sites as well as centers and family child care homes may help to explain differences between findings of our study and those of the Connecticut study.

To our knowledge, this is the first study to compare drinking water in CACFP and non-CACFP sites, and we found that more CACFP sites than non-CACFP sites had water available indoors and outdoors. CACFP sites have also been shown to serve healthier foods than non-CACFP, likely because of reimbursement for food costs and additional training (19). Unlike non-CACFP sites that are covered only by state water policy, CACFP sites in California are covered by both federal and state policy, which may contribute to their better policy implementation. CACFP sites also receive more monitoring and technical assistance than non-CACFP sites. We previously showed that CACFP sites were indeed more likely to know about child care beverage policy (22).

Although beverage policy appeared to have a positive impact on water access in sampled California child care sites, a third of sites in 2012 reported having drinking water available to children some of the time or rarely or never. The 2012 survey data were collected less than a year after federal and state policies were implemented, and therefore water provision may improve as providers gain familiarity with policy requirements. However, additional efforts may be needed to ensure policy implementation across all child care settings. Interestingly, child care providers reported few barriers to serving water. The main barriers cited included a perceived government rule against water with meals or snacks (even though no such rule exists), unavailability of water in some locations, and cost.

Ideally, young children should learn to quench their thirst with water instead of sugar-sweetened beverages. Some providers were concerned that children would “fill up” on water and drink less milk or eat less if served water at the table with meals or snacks. There is little evidence to support the idea that water replaces caloric intake in adults and no evidence among young children (3). Although the US Department of Agriculture does not prohibit serving water with meals or snacks as part of CACFP, its memorandum on CACFP water policy states, “caregivers should not serve young children too much water before and during meal times; excess water may lead to meal displacement, reducing the amount of food and milk consumed by the children” (19). Research is needed to examine the question of displacement of calories with water consumption.

Child care providers reported that parent and child education on serving water was more important than the need for their own training. However, providers may not know what they do not know. In a previous study we reported that in 2012 only 60% of providers were aware of beverage policy (22). Therefore, provider training in addition to parent training may increase knowledge about water policy and strategies. Indeed, one outcome of our study was a recently enacted California state policy to ensure that newly licensed child care providers receive nutrition training (23). This policy, which goes into effect in January 2016, amends child care licensing laws to ensure that an additional hour of training on childhood nutrition is added to the 15 hours currently required on preventive health and safety. Future efforts should ensure that existing child care providers also receive this training.

This study has several limitations. Findings might have been different if the survey had had a higher response rate or if the same sites had been followed from prepolicy to postpolicy. Because of the high turnover rate of child care sites and providers, a pre–post study design was not possible. In addition, our data rely on self-reported practices by child care providers and do not quantify what children actually consumed. Providers may have not have understood the meaning of self-serve and “easily and visibly available.” Possible bias or misreporting could not be verified without onsite observations. Finally, differences in findings between 2008 and 2012 may be due to other factors (eg, the national Drink Up campaign) besides policy.

We found that water access in California child care settings improved after the enactment of federal and state policies, yet there is a continuing need to determine optimal ways to provide water in child care settings (24). Empowering parents and providers through education and training may help ensure that children have access to water and also instill a lifelong habit of choosing water when thirsty before “empty calorie” beverages such as fruit drinks and other sugar-sweetened beverages. Reversal of the obesity epidemic depends on a reduction in calorie intake relative to caloric needs, and promoting water, which has zero calories, could be an effective strategy. Future studies should evaluate the impact of beverage policy in child care settings on children’s consumption patterns, caloric intake, and weight status. We also need to identify best practices for the implementation and monitoring of water access policy so that the intent of the law, reduced childhood obesity, is achieved.
